# Pathophysiology of ion channels in amyotrophic lateral sclerosis

**DOI:** 10.1186/s13041-023-01070-6

**Published:** 2023-12-15

**Authors:** Robin N. Stringer, Norbert Weiss

**Affiliations:** 1 Department of Pathophysiology, Third Faculty of Medicine, Charles University, Prague, Czech Republic; 2 Institute of Organic Chemistry and Biochemistry, Czech Academy of Sciences, Prague, Czech Republic; 3 Center of Biosciences, Institute of Molecular Physiology and Genetics, Slovak Academy of Sciences, Bratislava, Slovakia

**Keywords:** Amyotrophic lateral sclerosis, Motor neurons, Ion channels, Neuronal excitability, Neurodegeneration

## Abstract

Amyotrophic lateral sclerosis (ALS) stands as the most prevalent and severe form of motor neuron disease, affecting an estimated 2 in 100,000 individuals worldwide. It is characterized by the progressive loss of cortical, brainstem, and spinal motor neurons, ultimately resulting in muscle weakness and death. Although the etiology of ALS remains poorly understood in most cases, the remodelling of ion channels and alteration in neuronal excitability represent a hallmark of the disease, manifesting not only during the symptomatic period but also in the early pre-symptomatic stages. In this review, we delve into these alterations observed in ALS patients and preclinical disease models, and explore their consequences on neuronal activities. Furthermore, we discuss the potential of ion channels as therapeutic targets in the context of ALS.

## Introduction

Amyotrophic lateral sclerosis (ALS) stands as the prevailing and most severe motor neuron disease (MND), impacting an estimated 2 in every 100,000 individuals [1]. While ALS tends to exhibits a higher prevalence in young men compared to young women, the underlying cause of this sex-based susceptibility remains elusive. As individuals age, this sex disparity gradually diminishes. Disease onset typically occurs around the age of 40, reaching its peak between 70 and 80 years, followed by a sharp decline. Notably, familial ALS presents an average age of onset between 40 and 60 years, while sporadic ALS presents an average age range of 58–62 years [2]. The discovery of ALS is attributed to Jean-Martin Charcot in 1874, following an exhaustive study conducted from 1865 to 1869 [3]. Charcot documented distinct pathological features, including the anomalous appearance of descending axons in the lateral spinal cord, and the degeneration of corticospinal motor neurons. These findings gave rise to the term “lateral sclerosis”. Furthermore, the degeneration of spinal motor neurons, leading to denervation and subsequent muscle wasting, justified the label “amyotrophic” [4].

While clinical presentation of ALS can vary among patients, the Gold Coast criteria are instrumental in facilitating early diagnosis. These criteria are applicable in both clinical settings and clinical trials [5]. According to the Gold Coast criteria, a diagnosis of ALS is made when patients exhibit documented normal motor function, followed by a history of progressive motor impairment during clinical evaluation. Patients must exhibit dysfunction in both upper motor neurons and lower motor neurons in at least one body region, be it bulbar, thoracic, cervical, or lumbosacral. In cases where upper and lower motor neuron dysfunction coexist, they must be identified within the same body region, or lower motor neuron dysfunction should be identified in at least two distinct body regions. Motor dysfunction should be observed in one bulbar muscle, one thoracic muscle, or two limb muscles innervated by different nerves and roots. These abnormalities can be identified through electromyography or clinical observations. Upper motor neuron dysfunction can be characterised by the presence of at least one of the following: (i) Occurrence of pathological reflexes, including Babinski sign, Hoffman sign, snout reflex, or crossed adductor reflex; (ii) A decline in voluntary movement coordination attributed to upper motor neuron dysfunction, excluding Parkinsonian lower motor neuron-related factors; (iii) An increase in deep tendon reflexes within a clinically weakened muscle or in an adjacent muscle; (iv) An increase in velocity-dependent tone (spasticity). In contrast, lower motor neuron dysfunction is characterised by the presence of at least one of the following: (i) Electromyography findings that encompass signs of continuous nerve damage, such as positive sharp waves, fibrillation potentials, or fasciculation potentials, as well as indicators of long-term nerve damage, including large motor unit potentials with prolonged duration (motor unit instability and polyphasia serve as corroborating signs but are not conclusive evidence); (ii) Clinical evaluation revealing signs of muscle weakness and atrophy. In addition to these diagnostic criteria, clinical examinations must effectively rule out other diseases through various tests, including magnetic resonance imaging, nerve conduction studies, needle electromyography, studies of cerebrospinal fluid or blood, and any other necessary investigations.

Clinical manifestations of ALS are primarily contingent on the initial region of the body affected. However, over time, these manifestations tend to progress, leading to widespread loss of motor function and the eventual paralysis of certain muscles. This progression culminates in near-complete paralysis of muscles, ultimately resulting in a fatal outcome. It is important to note that other MNDs, such as progressive muscular atrophy and primary lateral sclerosis, could be re-diagnosed as ALS if both upper and lower motor neurons are affected. Furthermore, ongoing research in the field of MNDs has revealed significant overlap in pathological, clinical, and genetic characteristics with frontotemporal dementia. Consequently, these conditions have been categorized as part of a common disease spectrum (for an in-depth review see [6]). Currently, only four drugs have received FDA approval for the management of ALS, none of which can fully halt the progression of the disease but can extend life. These drugs include Riluzole, Edaravone, PB-TURSO, and Tofersen. Each target different aspects of the disease and has shown some promise in delaying ALS symptoms. However, most studies on these drugs and potential new drugs emphasise the importance of early treatment, underscoring the significance of early diagnosis [7, 8, 9, 10].

An increasing number of biomarkers have been employed to establish more precise diagnostic criteria, differentiating ALS from other MNDs [11]. However, the search for readily accessible biomarkers in ALS has encountered obstacles, primarily due to analytical limitations when dealing with complex samples like blood. Moreover, the creation of biomarker sets or the integration of multiple investigative modalities to enhance sensitivity has proven to be a challenging endeavour. The cerebrospinal fluid (CSF) stands out as a pivotal source for remnants of neuro-axonal damage and metabolic shifts in both healthy and deteriorating neurons, making it a promising avenue for ALS biomarkers research. Its relatively simple composition facilitates the detection of even minute amounts of molecules. Yet, the deteriorating physical condition of ALS patients, characterized by communication difficulties, limited mobility, and heightened vulnerability in advanced stages, diminishes the feasibility of invasive techniques such as lumbar puncture for CSF extraction, Additionally, performing MRI scans on advanced ALS patients becomes problematic due to respiratory challenges and the accumulation of secretions. Consequently, while CSF and imaging methods may be effective for initial diagnosis and prognosis, as ALS progresses, biomarkers derived from blood or urine may offer a more convenient approach for assessing the progression of the disease and evaluating therapeutic approaches. Currently, there exist several CSF and blood biomarkers capable of distinguishing ALS from other MNDs when analysed in combination, as no single biomarker can provide a complete differentiation. Examples of biomarkers found in CSF/blood at higher levels in ALS patients compared to patients with other MNDs include chitotriosidase, chitinase-3-like protein 1 and 2, high-sensitivity cardiac troponin, neurofilament light/heavy chains, and the ratio of phosphorylated to total tau protein [12]. In addition, a variety of miRNA biomarkers have been identified as either upregulated or downregulated in ALS patients, which, when used in conjunction with protein biomarkers, can significantly enhance discriminatory power [13].

While the pathogenesis of ALS remains a subject of intense investigation and is still not fully understood, it is evident that it is a complex disorder with a genetic component contributing to both the susceptibility and the progression of the disease, along with several hypothesised environmental influences [4, 14, 15]. Numerous environmental toxins have been associated with ALS incidence, but four environmental factors have shown the strongest associations with ALS development in population exposure studies. These factors include formaldehyde, manganese, zinc, and mercury (for a comprehensive review see [15]). In addition, there is evidence for increased likelihood of ALS in professional athletes [16] as well as in army veterans [17]. Familial ALS, accounting for 5–10% of patients, displays clear signs of inheritance and is linked to mutations in ALS-associated genes. However, the majority of patients fall under the category of sporadic ALS, with no clear family history of the disease. Thanks to the adoption of genetic analysis, hundreds of single-nucleotide polymorphisms (SNPs) have been identified in ALS patients. Nevertheless, these are rarely disease-causing mutations and are often challenging to distinguish from normal variations in the general population (The 1000 Genomes Project Consortium, 2015). Nonetheless, over 25 causative genes have been reported, of which *C9orf72* (chromosome 9 open reading frame 72), *SOD1* (superoxide dismutase 1), FUS (fused in sarcoma RNA-binding protein), *TARDBP* (TAR DNA-binding protein), *VCP* (Valosin containing protein), and PFN1 (Profilin 1) are implicated in about 60–70% of familial ALS cases and around 10% of sporadic ALS cases (for a comprehensive genetic ALS review see [18]). The exploration of genetically diverse cases of familial and sporadic ALS have identified a number of cellular alterations that precede or occur in parallel with the development of the disease. For instance, misfolded TAR DNA-binding protein-43 (TDP-43) aggregates are found in about 97% of all familial and sporadic cases. The remaining 3% of patients present SOD1 (around 2%) and FUS (< 1%) protein aggregates [19]. Additionally, alterations in RNA and RNA-binding protein levels, activation of non-neuronal cells such as neuroinflammatory cells (microglia and astroglia) and oligodendroglia, as well as structural and functional alteration of the neuronal cytoskeleton have been reported [20]. Furthermore, oxidative stress and mitochondrial dysfunction have been documented in the pathogenesis of ALS, likely caused by alterations in RNA and RNA-binding proteins (for an in-depth review see [21]). Likewise, dysfunctions in axonal transport [22, 23], ubiquitin–proteasome system [24], and nucleocytoplasmic transport [25, 26] are observed in the pathogenesis of ALS. Importantly, one of the most noticeable cellular hallmarks of ALS is the alteration of neuronal electrical activity.

In this review, we provide a succinct overview of these neuronal electrical alterations and subsequently delve into a comprehensive analysis of the underlying mechanisms, with a focus on the involvement of ion channels as key players in regulating neuronal excitability. Finally, we explore the pharmacological efforts initiated to therapeutically target ion channels in the treatment of ALS.

## Altered neuronal excitability in ALS

Changes in neuronal electrical activity constitute a defining characteristic of ALS. Initially, the observation that motor neurons become hyperexcitable as the disease progressed led to the belief that this heightened excitability was a response to the decline of spinal motor neurons [27]. However, subsequent research has revealed that this hyperexcitability actually occurs before any notable loss of spinal motor neurons [28, 29, 30, 31, 32, 33]. Furthermore, heightened cortical excitability is detected even before early clinical symptoms, such as fasciculations, hyperreflexia, cramps, and spasticity, become evident [29, 34]. This escalation in neuronal activity results in several adverse consequences, including changes in mitochondrial functions [35], disruptions in energy metabolism [36], and increased oxidative stress [37]. Different types of motor neurons react differently to these negative effects due to their intrinsic properties.

Motor neurons fall into three main groups: α-, β-, and δ-neurons. Notably, α-motor neurons are responsible for innervating extrafusal muscle fibers and play a crucial role in muscle contraction [38]. Their degeneration is believed to be the primary target related to ALS dysfunction. These α-motor neurons can be further categorized based on the extrafusal fibre they innervate, i.e. slow-twitch fatigue-resistant (SFR), fast-twitch fatigue-resistant (FFR), and fast-twitch fatigable (FF) fibers [39]. SFR motor neurons typically have smaller cell bodies, and therefore have higher input resistance, meaning they respond to lower synaptic activation, making them the first to be recruited for the initiation of muscle contraction. In contrast, FF motor neurons, possessing larger cell bodies, are recruited after SFR neurons, providing additional strength to muscle activation. When it comes to signal transmission speeds, motor neurons serving fast fibres are significantly quicker (100 m/s) than their SFR counterparts (85 m/s) [39]. As for FFR motor neurons, they lie in between FF and SFR motor neurons in terms of both soma size and signal transmission speed and strength. Larger motor neurons, like the FF and FFR types, which produce a greater number of action potentials and hence have increased energy and metabolic needs, might be more susceptible to damage and degeneration due to excitotoxicity [40, 41]. On the other hand, smaller motor neurons, such as the SFR type, seem to be more resilient, potentially offsetting the loss of other motor neurons, which can lead to a postponement in the onset of neurological symptoms [42].

**Fig. 1:**
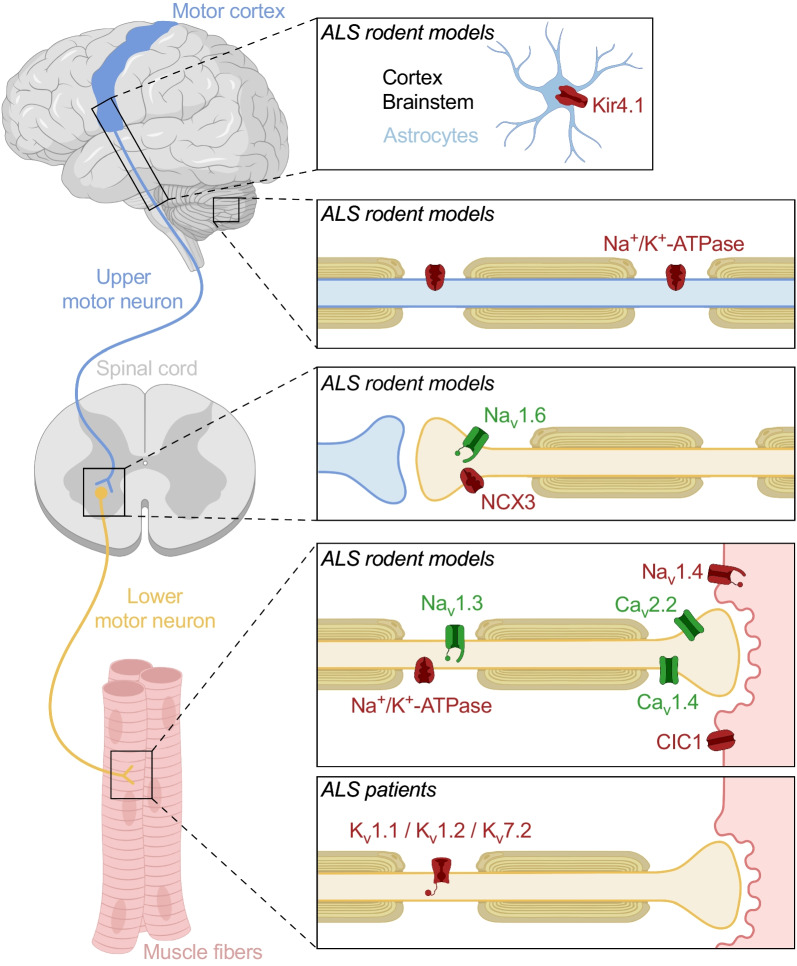
Alteration of ion channels along the motor pathway in ALS rodent models and patients. Channels shown in red are downregulated, while channels shown in green are upregulated. CIC1, chloride channel; Kir4.1, inward rectifier potassium channel; K_v_1.1/K_v_1.2/K_v_7.2, voltage-gated potassium channels, Na+/K+-ATPase, sodium/potassium ATPase; Na_v_1.3/Na_v_1.4/Na_v_1.6, voltage-gated sodium channels; NCX3, sodium/calcium exchanger

Three hypotheses have been proposed to explain the underlying mechanisms behind neuronal hyperexcitability: (i) defects in ion channels in ALS-associated neuronal and non-neuronal cells (Fig. 1; Table 1), (ii) dysfunction of cortical inhibitory circuits, and (iii) glutamate-mediated excitotoxicity [43]. In the next sections, we will briefly explore the effects of cortical inhibitory circuits and glutamate-mediated excitotoxity in causing hyperexcitability and then delve into a more detailed investigation of the role of ion channels in ALS, as this is the focal point of the review.

**Table 1:** Alterations of ion channels in ALS patients and preclinical rodent models

	Channel	Effect	Model	References
Hyperexcitability	Na_v_1.2	Increase in persistent sodium current	Application of PR_20_ on HEK-293 T cells expressing recombinant channel	[80]
	Na_v_1.3	Hyperpolarizing shift in voltage dependence of activation	Spinal motor neurons from SOD1^A4V^ mice	[76]
	Na_v_1.4	Reduction in mRNA expression	Skeletal muscle cells from the Tibialis Anterior muscle from SOD1^G93A^ mice	[93]
	Na_v_1.6	Increase in protein expression	AIS of spinal motor neurons from SOD1^G127X^ mice	[79]
	KCNQ2	Reduction in mRNA expression	Spinal motor neurons of ALS patients	[96]
	KCNA1	Reduction in mRNA expression	Spinal motor neurons of ALS patients	[96]
	KCNA2	Reduction in mRNA expression	Spinal motor neurons of ALS patients	[96]
	Kir4.1	Reduction in expression and activity	Cortex and brainstem astrocytes of SOD1^G93A^ rats	[98]
	Kir4.1	Reduction in expression and activity	Oligodendrocytes of the ventral horns of SOD1^G93A^ rat lumbar and cervical spinal cords and myelin fraction from the spinal cord	[100]
	NCX3	Reduction in expression and activity	Spinal motor neurons from SOD1^G93A^ mice	[106, 108]
	Ca_v_2.2	Increase in mRNA expression	Spinal motor neurons from SOD1^G93A^ mice	[112]
	Ca_v_1.4	Increase in persistent current	Spinal motor neurons from SOD1^G93A^ mice	[112]
	CIC-1	Reduction in mRNA expression	Skeletal muscle from SOD1^G93A^ mice	[93]
Hypoexcitability	Na/K-ATPase	Reduction in protein expression	Spinal cord and cerebellum from SOD1^G93A^ mice	[36]
	Ca_V_3.2	V1689M mutation—Depolarizing shift of the voltage dependence of activation	tsA-201 cells expressing recombinant channel	[132]
	Ca_V_3.2	A1705T mutation—Hyperpolarizing shift of the voltage dependence of steady state inactivation	tsA-201 cells expressing recombinant channel	[132]
	Ca_v_3.2	ΔI153 mutation—total loss of channel expression	tsA-201 cells expressing recombinant channel	[133]
	Ca_v_3.2	P1210L mutation—decreased T-type current and channel expression	tsA-201 cells expressing recombinant channel	[133]

### Dysfunction of cortical inhibitory circuits

Central nervous system (CNS) interneurons are key modulators of neuronal signalling. The majority of interneurons in the cortex are inhibitory, using neurotransmitters like γ-aminobutyric acid (GABA) or glycine. Among these, GABAergic interneurons have a profound impact on neuronal activity within the cortex. In healthy individuals, a minor stimulus to the motor cortex typically triggers inhibitory GABAergic interneurons, resulting in a reduction of subsequent neuronal activity, a phenomenon referred to as short interval intracortical inhibition (SICI) [44]. In contrast, ALS patients often exhibit a lack of SICI, suggesting potential dysfunction or loss of these inhibitory neurons [43]. Numerous studies have identified pre-symptomatic cortical hyperexcitability in various mouse models of ALS and ALS patients [33, 45, 46, 47]. For instance, one study found a reduction in spontaneous GABA release and GABAergic activities in the early stages of ALS in the wobbler mouse model [48]. Further investigations in a SOD1^G93R^ zebrafish ALS model revealed early signs of interneuron dysfunction, including a reduction in interneurons and inhibitory currents occurring before motor neuron defects [49]. In human studies, post mortem analysis of primary motor cortex tissue from ALS patients unveiled a downregulation in the expression of the GABA receptor subunit α and a loss of GABAergic interneurons [50]. Another study reported lower levels of GABA and a loss of SICI in ALS patients [46]. These findings strongly suggest compromised GABA signalling in both ALS models and patient samples. Additional research is necessary to elucidate whether the reduction in GABAergic signalling observed in these studies results from the downregulation of GABA receptors in motor neurons, a loss of GABAergic interneurons, or a combination of both factors.

### Glutamate-mediated excitotoxicity

Neuronal activity is regulated by pathways both within neurons and within the surrounding cells, such as glial cells. Astrocytes, a type of glial cell, play a crucial role in modulating neuronal activity by supporting the clearance of neurotransmitters from the synaptic cleft. The absorption of glutamate, a primary excitatory neurotransmitter, by astrocytes is vital to protect neurons from overactivation and its subsequent deterioration. It is believed that the heightened excitability in ALS patients arises from an excess of glutamate stimulating motor neurons. Several studies support this hypothesis, as they have detected an increase in glutamate levels in the CSF of ALS patients [51, 52]. Furthermore, a decrease in the expression levels of two excitatory amino acid transporters (EAAT1 and EAAT2) was discovered in post-mortem human ALS tissue [53]. These transporters are primarily expressed in astrocytes and are responsible for the uptake of glutamate from the synaptic cleft after neuronal activity. When neurons are exposed to excessive glutamate levels, it can induce their death by increasing calcium influx. Due to the limited calcium buffering capacity of motor neurons, a surge in calcium can activate various enzymes and disrupt mitochondrial function, ultimately leading to cell apoptosis [54]. Riluzole is believed to counteract this process not only by inhibiting persistent sodium currents but also by suppressing glutamate release and its subsequent response. Despite these findings, an intriguing hypothesis has emerged in recent years. This hypothesis posits that the increase in extracellular glutamate in ALS may actually be beneficial to motor neurons. It suggests that glutamate is used as a metabolite in ischemic neurons rather than as a neurotransmitter. This ischemia occurs when CSF accumulates, compressing the neurons and causing ischemia. However, proving this hypothesis is currently challenging due to the limited data on the CSF volume in ALS patients. Nonetheless, most of this theory is supported by evidence of spinal cord and cortical compression (for a comprehensive overview see [55]).

## Alteration of ion channels and neuronal hyperexcitability

While the majority of ALS patients do not exhibit noticeable deleterious mutations in the genes encoding ion channels, numerous studies have reported alterations in the expression and activity of several ion channels and transporters. Importantly, several mouse models carrying patient-associated mutations in ALS genes have provided valuable insights into the role of ion channels in ALS. Among these models, the SOD1 models have been extensively utilized. Although they display slight variations in disease development influenced by their genetic background [56], they all express high levels of mutated SOD1, leading to significant axonal denervation, motor neuron loss, increasing paralysis, and a reduced lifespan [57, 58, 59]. Another major model relies on mutations in TAR DNA-binding protein 43 (TDP-43), and a variety of transgenic have been generated [60]. These mice exhibit pathogenic aggregates of ubiquitinated proteins in certain neuronal populations and generally all display early onset neurological defects followed by significant motor dysfunction [61]. Similarly, mice carrying patient-associated mutations in FUS show protein aggregates, motor neuron degeneration, and eventual death [62]. Interestingly, the FUS models die much faster compared to the TDP-43 models, consistent with FUS mutations being associated with early-onset ALS [63]. While other models of ALS have been used to study the disease, these are the main three models referred in this review (for a comprehensive review of ALS models see [64]).

In the next sections, we explore the alterations of specific ion channel families and their consequences for neuronal excitability.

### Sodium channels

Alterations in sodium ion channels have been documented in ALS patients and several ALS mouse models. For instance, multiple studies have reported an increase in the strength-duration time constant, a measure of axonal excitability [28, 65], in ALS patients. This increase is associated with an increase in persistent sodium conductance [28, 65, 66, 67]. Investigations on the SOD1^G93A^ ALS mouse model have similarly reported an increase in persistent sodium currents in spinal and cortical motor neurons [30, 40, 68]. Remarkably, these changes have been observed even in pre-symptomatic animals, suggesting an early occurrence of increased persistent sodium currents before symptom onset, which then persist throughout the disease's progression. Persistent sodium currents are present in virtually all isoforms of voltage-gated sodium channels (Na_v_) and are influenced by factors such as extracellular calcium concentrations, oxygen concentration, alternative mRNA splicing, G-protein coupled receptors, and protein kinases [69, 70, 71, 72]. These currents feature low activation threshold, slow gating properties, and while they are generally small they persist over prolonged depolarization. In normal conditions, they facilitate repetitive firing and modulate membrane potential in the subthreshold range, thereby enhancing synaptic transmission [73]. Given these attributes, the increased persistent sodium currents likely contribute to the observed neuronal hyperexcitability in ALS [74].

In addition, alterations of the fast transient sodium current generated by Na_v_ channels have also been documented in spinal motor neurons from SOD1^G93A^ mice [75]. Transient sodium currents are responsible for the full depolarisation of excitable cells during action potentials. In spinal motor neurons from SOD1^G93A^ mice they showed a faster recovery from inactivation compared to wild-type spinal motor neurons, therefore promoting hyperexcitability [68]. Few studies however have assessed the molecular identity of the specific Na_v_ channel subtypes affected. Nonetheless, one of the few studies in SOD1^A4V^ transgenic mice reported an increase in total sodium current and a hyperpolarising shift in voltage dependence of activation of the Na_v_1.3 channel [76]. Yet, since Na_v_1.3 is primarily expressed in significant amounts in motor neurons at birth, diminishing to minimal levels in adulthood, its relevance to ALS pathology remains limited [77]. Considering that symptoms of ALS only manifest in adulthood, it becomes important to specifically assess Na_v_1.1, Na_v_1.2, and Na_v_1.6 channels, which are expressed at larger levels in adult motor neurons [78]. A recent study observed an increase in Na_v_1.6 protein expression levels in the ALS SOD1^G127X^ mouse model at the axon initial segment (AIS) of isolated spinal motor neurons, where they play a role in shaping the action potential before it propagates along the axon [79]. They also found an increase in hyperpolarization-activated currents and a decrease in the width of the AIS which suggests that the sodium channels in these regions are more tightly packed thereby increasing the excitability of AIS [79].

Furthermore, in mice treated with proline-arginine (PR) poly-dipeptides derived from the *C9orf72* repeat expansion linked to ALS, there was heightened excitability in motor cortex pyramidal neurons, likely through an increase in persistent sodium current primarily mediated by Na_v_1.2 channels [80]. The C9orf72 repeat expansion undergoes atypical translation to produce poly-dipeptides: poly-glycine-proline (GP), poly-glycine-alanine (GA), poly-glycine-arginine (GR), poly-proline-arginine (PR), and poly-proline-alanine (PA) [81, 82, 83, 84, 85, 86, 87]. Numerous research findings have highlighted the neurotoxic properties of poly-PR and poly-GR, primarily due to their interference with RNA biogenesis [81, 88, 89] and cell organelle structure and function [90]. Alterations in Na_v_ channels could stem from factors such as varying expression levels of α- and β-subunits, changes in channel gating properties, transcriptional modifications, modulation by endogenous signalling molecules, or indirect effector changes [75]. For instance, increased expression of the β_3_ subunit of Na_v_ channels has been observed in a mutant SOD1 mouse model of ALS at pre-symptomatic stages [91]. This increase in β_3_ subunit was reported to enhance neuronal firing around the excitability threshold via its effect on Na_v_ channels and as such can contribute to hyperexcitability [92]. The pre-symptomatic occurrence found in these studies support the idea that neuronal hyperexcitability is an early pathological sign of ALS and identifying the pathways that cause alterations in the activity of these channels may be useful in developing new therapeutic strategies.

Furthermore, in skeletal muscle cells isolated from the tibialis anterior muscle from 90- and 130-days old SOD1^G93A^ mice, a significant reduction in the expression of Na_v_1.4 mRNA was observed. Na_v_1.4 channels are responsible for fully depolarising muscle cells during an action potential. Thus, the decrease in mRNA could in part explain the decrease in action potential amplitude in these muscle cells [93, 94].

### Potassium channels

An increase in resting potassium conductance has been reported in fast-twitch flexor digitorum brevis muscle fibres isolated from an ALS mouse model [43]. This increase is believed to be linked to an increase in ATP-sensitive potassium channel (KATP) current, which was attributed to an increased expression of SUR1, an auxiliary subunit of the KATP channel complex. Under normal conditions, SUR1 modulates KATP channels by enhancing sensitivity to ATP and Mg-nucleotides, thereby increasing the opening probability of the channel [95]. Conversely, several other studies on ALS patients and animal models of ALS have documented a reduction in expression levels of various potassium channels [31, 66, 96, 97, 98]. For instance, reduced mRNA levels of *KCNQ2* encoding the voltage-gated potassium channel K_v_7.2, as well as mRNA levels of *KCNA1* and *KCNA2* encoding the voltage-gated potassium channels K_v_1.1 and K_v_1.2, respectively, were reported in spinal motor neurons of ALS patients [96]. Multiple studies have also reported a reduction in fast and slow potassium conductances in ALS patients by using external electrodes to measure the compound muscle action potential from the abductor pollicis brevis [31, 66, 97]. Given that potassium channels are usually responsible for counterbalancing the inward current propagated by sodium channels, their inhibition at the presynaptic side has been found to trigger spontaneous firing at the neuromuscular junction therefore increasing the likelihood of hyperexcitability in ALS affected neurons [99]. Another study reported a decrease in expression and activity of the inwardly rectifying potassium channel Kir4.1 in the cortex and brainstem of an ALS SOD1^G93A^ rat model [98]. These channels are primarily expressed in astrocytes and maintain the neuronal microenvironment, taking up excess potassium ions released after neuronal activity. This suggests that deregulation of potassium homeostasis in the blood brain barrier astrocytic lining may cause neuronal excitotoxicity, neuronal degeneration, and apoptosis in both neurons and astrocytes [98]. Moreover, a decreased expression in Kir4.1 was observed in oligodendrocytes of the ventral horns of SOD1^G93A^ rat lumbar and cervical spinal cords, as well as in myelin fraction from the spinal cord, along with a reduction of Kir currents in oligodendrocytes [100]. Additionally, healthy spinal motor neurons plated with ALS patients- or SOD1^G93A^-derived oligodendrocytes undergo cell death [101]. These findings suggests that the effects on inward current as well as the high level of mutant SOD1 aggregates found in ALS SOD1^G93A^ mouse spinal cords [102] play an important role in oligodendrocyte dysfunction and in turn neuronal survival in ALS pathology.

### Calcium channels

Calcium, a primary intracellular messenger, affects multiple processes such as synaptic plasticity and transmission, neuronal development, and regulation of some metabolic CNS pathways. Therefore, a rise in firing frequency associated with the various defects mentioned previously could increase calcium uptake into motor neurons via voltage-gated calcium channels [103]. Furthermore cortical, spinal, and lower cranial nerve motor neurons isolated from ALS patient’s post-mortem have reduced expression of calcium-buffering proteins such as calbindin and parvalbumin, potentially contributing to calcium excitotoxicity [104]. Consistent with this notion, an alteration of calcium homeostasis in spinal motor neurons of mice caused significant vulnerability to excitotoxicity mimicking what is observed in ALS-vulnerable motor neurons [105]. This excess of calcium build up causes chronic depolarisation of the mitochondrial membrane, which aids in the activation of proteins involved in pathways leading to apoptosis [106]. In contrast, ALS-resistant motor neurons, such as oculomotor neurons, display a five- to sixfold increase in calcium buffering when isolated from WT mice [107] indicating that calcium homeostasis is important in motor neuron degeneration.

Multiple calcium channels, including the Na^+^/Ca^2+^ exchanger (NCX) and plasma-membrane calcium ATPase, play roles in buffering calcium ions. The considerable decrease in expression and activity of NCX3 in spinal motor neurons isolated from SOD1^G93A^ mice results in an excess of mitochondrial calcium and ROS production [106, 108]. Several studies have proposed a critical role for NCX3 in mediating deterioration in neuromuscular transmission in neuronal disorders, including ALS [108, 109, 110, 111]. This could therefore be a new target for ALS treatment, as overexpression and activation of NCX3 was able to aid in ionic homeostasis during the progression of ALS, lessening motor neuron degeneration [108].

Furthermore, an increase in high-voltage-activated (HVA) calcium currents was identified in mouse SOD1^G93A^ spinal motor neurons in the early pathogenesis of ALS, without change in low-voltage-activated (LVA) calcium currents [112]. In motor neurons, voltage-gated calcium channels (VGCCs) are important in the initiation of action potentials and in modulating firing frequency [113]. The majority of the increase in HVA current was attributed to an increase in Ca_v_2.2 (N-type) calcium channel expression; however, increases were also found in mRNA expression of CACNA1A (Ca_v_2.1 channel, P/Q-type), CACNA1C (Ca_v_1.2 channel, L-type), and CACNA1E (Ca_v_2.3 channel, R-type) in spinal motor neurons of this SOD1^G93A^ mouse model [112]. This group also documented an increase in persistent calcium current through L-type calcium channels. This overall increase in activity of HVA channels suggests an increase in excitability of spinal motor neurons. Furthermore, there is evidence that L-type calcium channel antagonists saves cultured ALS mouse spinal motor neurons and dorsal root ganglia cells when the SOD1^G93A^ mutation was genetically transferred to cultured cells [114, 115]. Indicating a possible therapeutic target.

### Chloride channels

More recently, a reduction in CIC-1 chloride channel mRNA expression levels was reported in the skeletal muscles of the ALS SOD1^G93A^ mouse model [93]. Additionally, protein kinase-C (PKC), which is known to phosphorylate and inhibit the expression of CIC-1 [116], was found to be overexpressed, causing further inhibition of CIC-1 channels [93]. This particular channel is typically expressed in skeletal muscle and helps modulate resting membrane potential and excitability [117, 118]. In normal conditions, it maintains membrane chloride conductance during rest and keeps this conductance low during the initial phase of the action potential [116]. Therefore, when under expressed in this ALS mouse model, skeletal muscle cells were found to be hyperexcitable, resulting in muscle fibre death.

Altogether, these modifications in ion channels found in skeletal muscle cells in ALS models suggest the need to find drugs that target a broader range of ion channels. While the current most effective treatment, Riluzole, targets multiple ion channels, it only extends life by ~ 6 months. Since ALS is a multifactorial disease, addressing the root cause of hyperexcitability in various cell types of the motor unit may be the path to progress.

## Alteration of ion channels and neuronal hypoexcitability

In apparent contradiction to the prevailing theory of early hyperexcitability as a hallmark of ALS pathology, an alternative perspective proposing hypoexcitability has also emerged [119, 120, 121, 122]. For instance, MNs derived from human induced pluripotent stem cells (iPSCs) carrying either the TARDBP^M337V^ or the C9ORF72 mutations exhibit initial hyperexcitability that subsequently shifts towards hypoexcitable states at around 7–8 weeks in vitro [121]. However, it is essential to note that this study employed iPSCs generated from fibroblasts of ALS patients cultured in vitro, making direct comparison to MN cells developing in an in vivo context challenging. Nonetheless, this observation finds support in another study revealing that spinal motor neurons isolated from SOD1^G93A^ and FUS^P525L^ ALS mouse models shift towards hypoexcitability within the larger motor neuron pools, such as fast-fatigable and large fast fatigue-resistant types, potentially leading to neuronal degeneration [122]. A study using SOD1^G93A−low^ mice, which have a low expression of SOD1^G93A^, observed early hypoexcitability in the delayed-onset firing group of lumbar spinal motor neurons from P8-P9 mice. This was evidenced by a diminished firing frequency to current relationship and an elevated voltage threshold. The study suggests that these delayed-onset firing motor neurons may correspond to FF motor neurons. However, there is no definitive proof to support this assertion. If accurate, it would indicate that ALS-sensitive motor neurons, like FF motor neurons, manifest hypoexcitability early in ALS disease progression. On the other hand, in the SOD1^G93A−high^, where there is a high expression of SOD1^G93A^, hyperexcitability was observed in the sustained firing subset of lumbar spinal motor neurons at the same developmental stage [123]. The study did not clarify which α-motor neuron subset this pertains to. Yet, it is intriguing to note that, based on the SOD1^G93A^ expression level, various subsets of lumbar spinal motor neurons exhibit either hypoexcitability or hyperexcitability at an identical developmental stage.

A growing number of studies [119, 122, 124] has found that hypoexcitability is an early feature of ALS, primarily observed in the largest motor neurons that innervate FF muscle fibres, followed by the large motor neurons that innervate FFR muscle fibres. This occurs before motor units start to degenerate. One possibility for this is that other more resistant motor neurons compensate for the reduced activity of these hypoexcitable motor neurons. Another plausible explanation for this hypoexcitability suggests that ALS-affected neurons initially become hyperexcitable due to transcriptional changes triggered by ALS mutations. Subsequently, these cells might attempt to compensate for this hyperexcitability through mechanisms that are currently not well understood [125]. This overcompensation could then lead to a state of hypoexcitability, ultimately contributing to neuronal degeneration [71]. Hypoexcitability could instead serve to reduce calcium uptake, thereby increasing motor neuron survival [119]. Given that the discovery of motor neuron hypoexcitability in ALS patients is relatively recent, the underlying mechanisms are not yet well-documented. However, in the subsequent sections, we delve into the alterations in ion channels that might contribute to this state of neuronal hypoexcitability.

### ***Na***^+^***/K***^+^***-ATPase***

A imbalance in both sodium and potassium ions was discovered in the spinal cords of transgenic mutant SOD1^G39A^ mice, attributed to a significant loss in ouabain-sensitive Na^+^/K^+^-ATPase, which was also observed in the cerebellum [36]. In normal circumstances, this pump is responsible for maintaining low cytosolic sodium levels and high cytosolic potassium levels using ATP. In neurons, this function allows them to return to their resting state after an action potential, while in astrocytes, the established sodium gradient facilitates neurotransmitter uptake [126]. This suggests that neurons do not recover from activation as quickly, suggesting a decrease in excitability. Furthermore, the Na^+^/K^+^-ATPase consumes around 50% of the energy supply within the CNS [127]. A loss of more than 75% in Na^+^/K^+^-ATPase activity will significantly affect the energy metabolism of the cells, potentially offsetting the pathological effects of mitochondria known to occur in ALS pathology. This highlights the necessity for further investigation [128]. The Na^+^/K^+^-ATPase consists of two subunits, α and β. Interestingly, a significant decrease in α-subunits was detected in spinal motor neurons isolated from SOD1^G93A^ mice, with no effect on β-subunits [36]. This loss of α-subunits can be explained by their increased sensitivity to damage by free radicals and other oxidative stress [129]. This sensitivity arises because a substantial portion of the α-subunit faces the reducing environment of the cytoplasm and has 23 free sulfhydryls and other oxidative groups that are readily oxidized. As such they are adapted to a reduced environment and are therefore easily oxidized by free radicals present in the cytoplasm. Oxidized Na^+^/K^+^-ATPase α-subunits can then be broken down by proteosomal, calpain, and lysosomal pathways [130, 131]. In contrast, β-subunits reside in the extracellular space where the environment is more oxidizing than the cytoplasm, and has six extracellular sulhydryl groups linked via disulfide bonds [36]. Thus, they are more adapted to an oxidizing environment and are less affected by free radicals. Due to the importance of this pump in maintaining correct neuronal signalling and energy metabolism, the Na^+^/K^+^-ATPase should be further explored as a potential therapeutic target.

### Calcium channels

In addition, another study reported two loss-of-function missense mutations in the gene *CACNA1A* encoding the pro-excitatory voltage-gated Ca_v_3.2T-type Ca^2+^ channel, in a patient with ALS [132]. The V1689M mutation produced a depolarising shift in voltage dependence of activation of the channel, suggesting that stronger depolarisations are necessary to activate the channel. The A1705T mutation produced a hyperpolarized shifted the voltage dependence of inactivation, suggesting decreased channel availability at the resting membrane potential of neurons. Likewise, two additional mutations in the Ca_v_3.2 channel, identified in an ALS patient, underwent functional analysis, confirming a similar loss-of-function. One mutation caused an in-frame deletion of a highly conserved isoleucine residue, leading to a complete loss of channel function. Moreover, this mutation exhibited a dominant-negative effect on the wild-type channel. The second mutation, in contrast, induced a milder reduction in the T-type calcium current [133]. These channels are expressed throughout the CNS and PNS mediating a low voltage activated, transient Ca^2+^ current that plays a crucial role in modulating neuronal excitability [134]. They have been documented in mouse spinal motor neurons though their specific role and the result of these loss-of-function mutations in the context of ALS remains to be further investigated [112].

Furthermore, T-type channels are known to modulate the activity of other ion channels, including calcium-activated and voltage-gated potassium channels. These functional interactions indirectly affect neuronal excitability through these signalling channel complexes [135]. Finally, six variants in *CACNA1D* encoding the L-type Ca_v_1.3 calcium channel were identified in ALS patients. These VGCCs are high voltage activated calcium channels that are primarily located in the post-synapse of neurons, but are also expressed in the sinoatrial node and atrial cardiomyocytes where they help modulate cardiac pacemaker activity. They are also found in the pancreas and kidney where they play a role in endocrine secretion and in hair cells of the inner ear where they modulate synaptic transmission [136]. From the six variants found in this channel one caused a loss-of-function of the channel, the functional effects of the other variants and there potential implication in ALS are yet to be assessed [137]. Nonetheless, it is possible that loss-of-function of voltage-gated calcium channels may indeed contribute to the hypoexcitability reported more recently in specific motor neurons [119].

## Targeting ion channels ALS

So far, the Food and Drug Administration (FDA) had granted approval to four drugs for the management of ALS. These drugs include Riluzole [138], Edaravone [139], PB-TURSO [140], and Tofersen [141], Riluzole is the sole drug primarily designed to target ion channels. Nevertheless, various other ion channel modulators have been evaluated or are currently under investigation (Table 2).

**Table 2:** Drugs targeting ion channels currently in use or in trials for ALS

Drug	Structure	Target	Effect	Stage	References
Riluzole		Na^+^ channels	Reduces persistent Na^+^ current	In use	[30]
		K^+^ channels	Activates channels and inhibits slow inactivation of voltage-gated K^+^ current		[143, 144]
		Ca^2+^ channels	Inhibits persistent and transient Ca^2+^ currents		[9, 40, 145, 146]
Gabapentin		Voltage-gated Ca^2+^ channels	Inhibits channel expression	Phase III	[151]
Pimozide		T-type Ca^2+^ channels	Inhibits Ca^2+^ currents	Phase II	[154]
FPL 64176		L-type Ca^2+^ channels	Activates channels	Preliminary testing	[155]
Bay K 8644		L-type Ca^2+^ channels	Activates channels	Preliminary testing	[155]
Mexiletine		Na^+^ channels	Inhibits persistent Na^+^ current	Phase IV	[156]
Ezogabine		K^+^ channels	Activates channels	Phase II	[161, 162]
QRL-101		K^+^ channels	Activates channels	Phase I	[163]
4AP		K^+^ channels	Inhibits K^+^ currents	Preliminary testing	[120]

### Riluzole

Riluzole was approved in 1995 and is believed to exert its neuroprotective effects through several mechanisms: (i) Reducing persistent sodium current and firing frequency in ALS neurons back to control levels [30]; (ii) Inhibiting glutamate release and increasing glutamate uptake [142]; (iii) activating various types of potassium channels and inhibiting slow inactivation of voltage-gated potassium channels, thus reducing spontaneous firing and hyperexcitability [143, 144]; and (iv) Inhibiting persistent calcium currents [9, 40, 145] and transient calcium currents [146]. The interplay between reduced potassium conductance and increased persistent sodium currents is considered the primary driver of motor neuron hyperexcitability in ALS patients. This heightened excitability leads to increased firing frequency, resulting in characteristic early symptoms like fasciculations and muscle cramping [11]. However, besides Riluzole, drugs targeting these two symptoms in animal models have shown no significant effects in clinical testing [147]. Importantly, combining Riluzole with other drugs has demonstrated improved efficacy, highlighting the potential of combination therapy in addressing ALS [148, 149]. This supports the notion that drugs targeting multiple ion channels might be effective due to the multifactorial nature of ALS. It is plausible that each ALS patient has a distinct subset of ion channels affected by the disease, contributing to the ALS condition. This may explain the limited extension of lifespan with Riluzole treatment alone, suggesting that a more targeted approach could yield better results. Alternatively, broad-spectrum ion channel modulators, like Riluzole, may not sufficiently address the specific ion channels that need normalization, potentially leading to deregulation and unwanted effects.

### Calcium channel modulators

Several drugs targeting voltage-gated calcium channels have been assessed for the management of ALS, although their effectiveness in slowing down ALS symptoms has been limited [147, 150]. One such drug tested in clinical trials is Gabapentin [151]. Gabapentin primarily acts on the α_2_δ ancillary subunit of voltage-gated calcium channels, inhibiting the expression of the channels in the plasma membrane [152]. However, a study found that Gabapentin only inhibits α2δ when overexpressed [153]. This might explain its lack of efficacy in ALS clinical trials, potentially indicating the need to test other inhibitors targeting different subunits including the calcium channel itself.

Hence, pimozide, a T-type calcium channel blocker, has recently shown potential in safeguarding neurons vulnerable to ALS. In an mTDP-43 zebrafish ALS model, pimozide successfully preserved the structure and transmission of the neuromuscular junction (NMJ) during repeated stimulations. Similarly, in the SOD1^G37R^ mouse model, pimozide effectively enhanced NMJ synaptic transmission. A brief phase II randomized controlled trial of pimozide spanning 6 weeks yielded encouraging outcomes, with ALS patients retaining better neuronal responses than the control group [154]. It is now essential to extend this trial over a more extended period and include a larger participant group to ascertain the long-term effects.

Additionally, L-type calcium channel agonists are currently under examination for their potential to rejuvenate motor neuron functionality. In an mTARDBP zebrafish ALS model, there was a restoration of the disrupted synaptic transmission upon using the L-type calcium channel agonists FPL 64176 and Bay K 8644. The data implies that the acute administration of FPL 64176 can recover swim duration, even if it does not enhance the distance or speed. This hints at its potential therapeutic value during advanced stages of the disease. However, the research also identified that the best time for treatment with these agonists is during the preclinical stages, and only at low concentrations. When used at higher dosages, both compounds exhibited toxicity [155].

### Sodium channel modulators

Mexiletine, a sodium channel blocker, is currently in phase 4 clinal [156]. Mexiletine has demonstrated its effectiveness in reducing muscle cramps in ALS patients [100], a symptom that afflict 90% of cases [157]. Mexiletine, akin to Riluzole, inhibits persistent sodium currents [158]. Interestingly, Riluzole was found to have no discernible impact on alleviating muscle cramps in ALS patients, and the combination of both Riluzole and Mexiletine showed no synergetic effect compared to Mexiletine alone [156, 159]. However, the utility of Mexiletine for the treatment of ALS is limited since it primarily targets muscle cramps, with some sporadic side effects at low doses that become more prevalent at higher doses [156].

### Potassium channel modulators

Ezogabine, also known as retigabine, is an antiepileptic drug that targets hyperexcitability in ALS patients. Ezogabine activates voltage-gated potassium channels, which could counteract the reduction in mRNA expression of *KCNQ2* (K_v_7.2 channel) observed in spinal motor neurons in ALS patient, as mentioned previously [96]. Furthermore, high-throughput screening with GCaMP, measuring calcium ion dynamics indicative of neuronal activity in human ALS cells, highlighted the K_v_7 ion channel as a key target for mitigating excitotoxicity [160]. In vitro studies showed that Ezogabine reduced hyperexcitability and extended the survival of SOD1^A4V/+^ ALS neuro and also reduced hyperexcitability in cortical and spinal MNs, thereby reducing neuronal firing to normal levels in a phase 2 trial [161, 162]. However, these findings are based on a small study of 65 patients with a short treatment duration, and the lack of selectivity Ezogabine was associated with frequent adverse effects. Therefore, QurAlis has developed a new drug, QRL-101, a more selective activator of K_v_7 potassium channels, which is currently in clinical trials [163].

Recently, 4-aminopyridine (4AP), a potassium channel blocker, was assessed as a mean to counteract neuronal hypoexcitability on FUS and SOD1 mutant iPSC-derived MNs [120]. 4AP restored normal spontaneous firing activity and synaptic input in MNs by inhibiting potassium currents, thereby preventing MN degeneration. This approach somewhat contrasts with the use of potassium channel activators such as Ezogabine. Since hypoexcitability typically appears later in the course of the disease and only when symptoms are evident – unlike hyperexcitability, which precedes ALS symptoms – combining both types of drugs in a stage-dependent manner might be the most effective strategy for addressing ALS.

## Conclusion

In conclusion, in this review we provided a comprehensive exploration of the intricate role of various ion channels in the pathology of ALS, shedding light on their complex involvement in the disease process. One of the intriguing aspects of ALS is the dynamic interplay between hyperexcitability and hypoexcitability of motor neurons. In the early stages of the disease, motor neurons often exhibit hyperexcitability, meaning they are overly active. This hyperexcitability may subsequently be compensated for, leading to a shift towards hypoexcitability, where the neurons become less active, just prior to neurodegeneration. This duality of excitability states adds complexity to our understanding of ALS. However, a more nuanced perspective emerges when considering the diversity of neuronal cell types impacted in ALS. It is plausible that hyperexcitability and hypoexcitability could manifest as distinct pathological characteristics within these neuronal subpopulations, contributing to the complex spectrum of ALS manifestations observed in patients.

Furthermore, ALS may encompass a spectrum of different pathologies affecting motor function. Some patients may exhibit early signs of hyperexcitability, while others experience hypoexcitability. This targeting of different groups of motor neurons contributes to motor neuron loss and disease progression. The wide range of symptom development and variable involvement of upper and lower motor neurons further supports this idea. However, interpreting these distinct groups of pathologies becomes challenging, particularly considering the prominent use of the SOD1 mutant models for ALS research. Ethical considerations prohibit direct sampling from live ALS patients, making alternative animal models essential for distinguishing between potential groups of ALS pathologies. These models could help uncover the diverse mechanisms underlying ALS and contribute to more targeted therapeutic strategies. Nonetheless, the existing medication, Riluzole, which targets multiple ion channels, reinforces the importance of ion channels in disease development.

Regrettably, the divergence in excitability results and the limited success of drugs targeting hyper- or hypo-excitability make it difficult to draw definitive conclusions about the efficacy of these therapeutic approaches. Consequently, identifying specific patient groups with ion channel defects and associated excitability imbalances could provide a promising avenue for developing more effective treatments. In the ongoing pursuit of unraveling the intricate complexities of ALS, further research is imperative. A deeper understanding of the specific ion channels involved, their interactions, and their contribution to the dual phenomena of hyperexcitability and hypoexcitability will be crucial for advancing our knowledge and improving therapeutic interventions.

## Data Availability

All data generated or analyzed during this study are included in this published article.

## Abbreviations

4AP: 4-Aminopyradine
AIS: Axon initial segment
ALS: Amyotrophic lateral sclerosis
C9orf72: Chromosome 9 open reading frame 72
Ca_v_: Voltage-gated calcium channels
CIC: Chloride channel
CNS: Central nervous system
CSF: Cerebrospinal fluid
EAAT: Excitatory amino acid transporters
fALS: Familial ALS
FF: Fast-twitch fatigable
FFR: Fast-twitch fatigable-resistant
FUS: Fused in sarcoma RNA-binding protein
GA: Glycine-alanine
GABA: γ-Aminobutyric acid
GP: Glycine-proline
GR: Glycine-arginine
HVA: High-voltage activated
iPSC: Induced pluripotent stem cells
KATP: ATP-sensitive potassium channel
Kir: Inwardly rectifying potassium channel
K_v_: Voltage-gated potassium channel
LMN: Lower motor neuron
LVA: Low-voltage activated
MND: Motor neuron disease
Na^+^/K^+^-ATPase: Sodium/potassium ATPase
Na_v_: Voltage-gated sodium channels
NCX: Na^+^/Ca^2+^ exchanger
PA: Proline-alanine
PFN1: Profilin 1
PKC: Protein kinase C
PR: Proline-arginine
sALS: Sporadic ALS
SFR: Slow-twitch fatigue-resistant
SICI: Short interval intracortical inhibition
SNP: Single-nucleotide polymorphism
SOD1: Superoxide dismutase 1
SUR1: Sulfonylurea receptor type-1
TARDBP: TAR DNA-binding protein
TDP-43: TAR DNA-binding protein-43
VCP: Valosin containing protein
